# Hydrogel derived from decellularized pig small intestine submucosa boosted the therapeutic effect of FGF-20 on TNBS-induced colitis in rats via restoring gut mucosal integrity

**DOI:** 10.1016/j.mtbio.2025.101783

**Published:** 2025-04-20

**Authors:** Minmin Wang, Dingwei Li, Shenyuan Ouyang, Bingjie Tong, Yumo Chen, Bingyu Ding, Jie Wang, Zhijiang Jiang, Helin Xu, Sunkuan Hu

**Affiliations:** a Department of Gastrointestinal Surgery Nursing Unit, Ward 442, The First Affiliated Hospital of Wenzhou Medical University, Wenzhou City, Zhejiang Province, 325000, China; b Department of Pharmaceutics, School of Pharmaceutical Sciences, Wenzhou Medical University, Wenzhou City, Zhejiang Province, 325035, China; c Department of Gastroenterology, the First Affiliated Hospital of Wenzhou Medical University, Wenzhou City, Zhejiang Province, 325000, China

**Keywords:** Ulcerative colitis, Fibroblast growth factor 20, Decellularized small intestinal submucosa, Hydrogel, Mucosal repair, Barrier integrity

## Abstract

Ulcerative colitis (UC) is a chronic inflammatory bowel disease characterized by impaired intestinal mucosal barrier function, leading to persistent inflammation and tissue damage. Current therapies often fail to address barrier dysfunction, highlighting the need for innovative treatments. This study developed a novel therapeutic strategy by combining decellularized porcine small intestinal submucosa (D-SIS) with fibroblast growth factor 20 (FGF-20) to promote mucosal repair and restore barrier integrity in a TNBS-induced colitis rat model. The D-SIS-based hydrogel, supplemented with hyaluronic acid (HA), was designed to enhance FGF-20 stability and enable sustained drug release. Results showed that the FGF-20-loaded hydrogel (MAF) exhibited excellent rheological properties, erosion resistance, and controlled drug release, making it suitable for rectal administration. *In vitro* cell experiments demonstrated that MAF enhanced Caco-2 cell proliferation, migration, and tight junction protein expression, restoring epithelial barrier integrity. In the colitis model, MAF significantly reduced disease activity index (DAI) scores, attenuated inflammation, and restored mucosal morphology. Additionally, MAF promoted goblet cell regeneration, enhanced mucus secretion, and upregulated intestinal stem cell markers, indicating its ability to repair both epithelial and mucus barriers. In conclusion, the MAF hydrogel represents a promising therapeutic approach for UC by combining the regenerative properties of FGF-20 with the bioactive support of D-SIS.

## Graphical abstract

**Figure:**
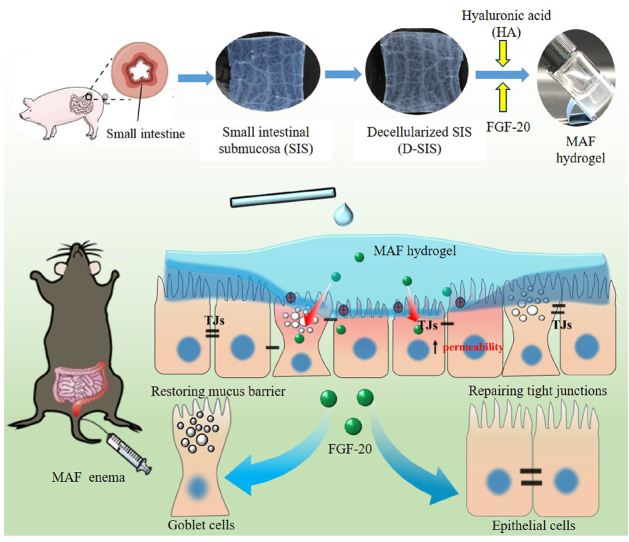

## 1 Introduction

Ulcerative colitis (UC) is a chronic inflammatory bowel disease (IBD) primarily affecting the colon and rectum, with symptoms including abdominal pain, diarrhea, and bloody stools [1]. Over recent decades, the global prevalence of UC has steadily increased, significantly impacting patients' quality of life and straining healthcare systems worldwide [2]. UC arises from a complex interplay of factors, including immune dysregulation, genetic susceptibility, gut microbiota imbalances, and compromised intestinal barrier function [[3], [4], [5]]. Among these, the breakdown of the intestinal mucosal barrier is recognized as a pivotal factor in UC development and progression [[6], [7], [8]]. The intestinal mucosal barrier acts as a vital defense system, preventing harmful substances such as pathogens and toxins from entering the bloodstream [9]. In UC, barrier dysfunction increases intestinal permeability, triggering immune activation and sustaining chronic inflammation [10]. This creates a vicious cycle where barrier damage and immune dysregulation perpetuate disease severity [11]. Despite advancements in understanding UC pathogenesis, current treatments remain limited. Standard therapies, such as 5-aminosalicylic acid analogs, immunosuppressants, and biologics, primarily focus on symptom control and immune suppression rather than repairing the damaged mucosal barrier [[12], [13], [14]]. These approaches often provide only temporary relief and are associated with challenges such as inconsistent efficacy, significant side effects, long-term dependency, and drug resistance [15]. As a result, there is a pressing need for innovative therapies that specifically target barrier repair, offering enhanced efficacy and minimized adverse effects.

Growth factors are signaling molecules that play critical roles in cell proliferation, differentiation, and tissue repair. In the context of intestinal diseases, particularly ulcerative colitis (UC), growth factors have emerged as promising therapeutic agents due to their ability to promote mucosal healing, enhance epithelial barrier function, and modulate immune responses. Among these, fibroblast growth factor 20 (FGF-20) has garnered significant attention for its reparative and anti-inflammatory properties [[16], [17], [18], [19]]. For example, in a mouse model of indomethacin-induced colitis, FGF-20 promoted epithelial cell proliferation, accelerated mucosal healing, and reduced inflammation [20,21]. These effects are mediated through its ability to enhance epithelial barrier integrity and modulate local immune responses, making it a promising candidate for UC treatment. Despite their potential, the clinical use of growth factors, including FGF-20 in UC faces several challenges. Their poor stability, low delivery efficiency, and potential to trigger immune responses limit their widespread use in intestinal diseases [22].

In recent years, tissue engineering has emerged as a promising approach for the treatment of ulcerative colitis (UC), offering innovative strategies to promote mucosal repair and regeneration. Among these, decellularized matrix (ECM) have gained significant attention as natural three-dimensional matrices that retain the structural and bioactive properties of native tissues [23,24]. These scaffolds provide mechanical support for cell growth and serve as delivery platforms for bioactive factors, making them ideal for regenerative medicine applications. Moreover, ECM components, such as heparan sulfate proteoglycans, collagens, and fibronectin, interact with growth factors to modulate their signaling and function [25,26]. This interaction is particularly important for delivery of growth factors, as it enhances their stability and ensures localized and sustained release at the site of tissue injury [27]. Porcine small intestinal submucosa (SIS) is a decellularized biomaterial derived from the jejunum, known for its translucent appearance and rich ECM components such as collagens, glycosaminoglycans, proteoglycans, and growth factors [[28], [29], [30]]. Decellularized SIS (D-SIS) has demonstrated significant potential in the treatment of UC, particularly in preclinical models. For example, it has been shown that D-SIS can effectively restore intestinal mucosal barrier function, attenuate inflammatory responses, and promote tissue repair in a dextran sulfate sodium (DSS)-induced colitis model [8].

**Fig. 1:**
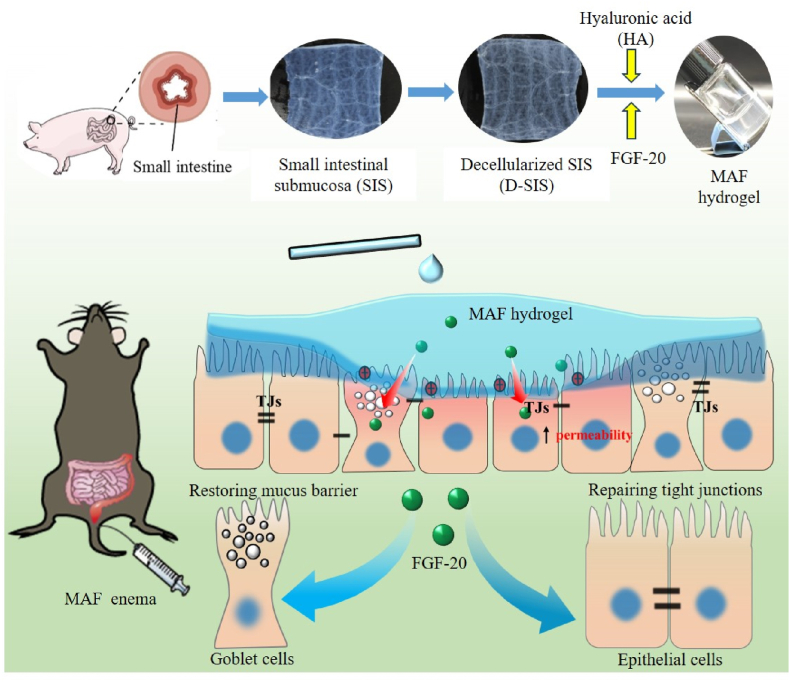
The schematic illustration of MAF hydrogel as a mimetic mucus restore gut mucosal integrity of colitis rats.

In this study, we developed a novel therapeutic strategy by combining decellularized porcine small intestinal submucosa (SIS) with fibroblast growth factor 20 (FGF-20) to promote mucosal repair and restore the intestinal mucus barrier of colitis rats (Fig. 1). The approach leverages the bioactive properties of D-SIS and the regenerative potential of FGF-20 to address the multifactorial pathology of UC. Hyaluronic acid (HA), a key component of the extracellular matrix (ECM), replicates the viscoelastic properties of intestinal mucus, providing a protective barrier for the damaged mucosa. To create a biomimetic scaffold, HA was incorporated into D-SIS to form an injectable hydrogel for deliver FGF-20. This hydrogel (MA) was expected as biomimetic intestinal mucus to enhance bioactivity of FGF-20. The physicochemical properties of MA hydrogel were comprehensively evaluated by rheologic test. *In vitro* release of FGF-20 from hydrogel was measured by shaking method and the interaction of MA with FGF-20 was also investigated by surface plasmon resonance analysis. *In vitro* cells experiment confirmed that MA significantly enhanced the proliferative activity of FGF-20. The therapeutic effect of FGF-20-loaded MA hydrogel (MAF) on colitis in rats was also investigated by evaluating the structural and functional integrity of the intestinal mucosa, reducing inflammation and promoting tissue regeneration.

## 2 Materials and methods

### 2.1 Materials and regents

Fresh porcine small intestine was obtained from the local butchery market. Recombinant human FGF-20 was purchased from Shanghai Universal Biotech Co.,Ltd (Shanghai, China). Hyaluronic acid (HA, 100 kDa) was provided by BOSF trading Co., Ltd (Hongkong, China). Dextran sulfate sodium (DSS, 40 kDa) was obtained from Yeasen Biotechnology Co., Ltd. (Shanghai, China). Pepsin (>3000 U/mg) was purchased by Sigma (Shanghai, China). CCK-8 kit were purchased by Solarbio Science &Technology Co. Ltd. (Beijing, China). IL-6, IL-1β, TNF-α and IL-10 ELISA kits were purchased from Jianglai Biochemical Technology (Shanghai, China). Decellularized pig small intestine submucosa (D-SIS) was prepared and characterized by the previous methods in our study [31].

### 2.2 Preparation of MAF hydrogel

Decellularized pig small intestine submucosa (D-SIS) hydrogel was prepared by pepsin digest method with some modification in previous study [32]. Briefly, the dry D-SIS was cut into small pieces and further milled into fine powder on Servicebio Tissues Milling. D-SIS powder (20 mg) was dispersed in 1 mL pepsin solution (1 mg/mL in 0.1 M HCl) and digested at 37 °C under shaking. Until the solid D-SIS granules were completely digested, pH of D-SIS solution was adjusted to 7 by addition of NaOH solution (0.1 M). Afterward, 0.1 mL FGF-20 solution (100 μg/mL) was added to the viscous D-SIS solution and mixed at 4 °C under gentle stirring. Finally, HA solution (20 mg/mL) was equally mixed with D-SIS-FGF-20 solution at 4 °C under gentle stirring to prepare MAF hydrogel. The blank hydrogel (MA) was also prepared by the similar procedure without addition of FGF-20.

### 2.3 Rheologic test and scanning electronic microscopy

The viscosity of hydrogel was detected by TRILOS rheometer (Anton Paar, USA) at the shear rate of 0.1–100 s^−1^. The rheologic behavior of the hydrogel were measured on DHR-2 rheometer (TA, USA) with cone plate (diameter of 40 mm). Oscillation-strains sweeps and oscillation-time sweeps were conducted at an angular frequency of 10 rad/s. Oscillation-frequency sweeps was measured at 1 % strain. Step-strain measurements was conducted at 100 % strain and 0.1 % strain, respectively. All rheologic tests were performed at 37 °C.

For SEM observation, hydrogel was frozen in liquid nitrogen, cross-sectioned and dried in vacuum. After sputtering a thin gold layer, the dried hydrogel was observed by SEM apparatus (HITACHI S-800, Japan) at 20 kV of voltage.

### 2.4 Surface plasmon resonance analysis

The binding of D-SIS with FGF-20 was analyzed by surface plasmon resonance (SPR). FGF-20 was immobilized on surface of GM5 sensor chip under EDC/NHS catalysis according to the method in previous study [33]. The dilute D-SIS solution flowed through the sensor chip at a flow rate (30 μL min^−1^) and the refractive index was recorded by an SPR instrument (GE Healthcare, Biacore T200, USA). The sensor outputs were analyzed by BIA evaluation software. A series of gradient D-SIS solutions were tested. Heparin solution was also tested as positive control.

### 2.5 *In vitro* dilution-resistant test and *in vitro* release experiment

*In vitro* erosion of MAF hydrogel was performed by the typical shaking method with some modification in previous study [31]. Briefly, hydrogel sample (1 mL) were added to the bottom of a vial. Afterward, artificial colon fluid (ACF, 4 mL) was added to cover hydrogel sample. The vial was shaken at a speed of 10 rpm in a thermostatic bath with 37 °C. At each time point, the residual hydrogel was measured and the percentage was calculated by the method in previous study [31].

*In vitro* release of FGF-20 from MAF hydrogel was also performed by the described method above. Alexa Fluro@647 was used to label the naïve FGF-20 and the fluorescent FGF-20 was loaded into MA hydrogel for *in vitro* release test. MAF hydrogel sample (1 mL) was placed on the bottom of a vial, followed by addition of the fresh release medium (ACF, 2 mL) to cover hydrogel sample. In vitro release test was performed at shaking speed of 10 rpm in a thermostatic bath with 37 °C. At each time point, all release medium in supernatant‌ was withdrawn and equivalence of fresh medium was supplemented at the same time. The released FGF-20 was detected by fluorescence spectrometer (Edinburgh Instruments,FS5) at excitation wavelength of 650 nm and emission wavelength of 670 nm. The fluorescent intensity was expressed as the cumulative release percentage.

### 2.6 *In vitro* cell experiments

Caco-2 cells were provided by FuHeng Biology (Shanghai, China) and cultured in DMEM medium (Gibco, USA) supplemented with 10 % fetal bovine serum and 1 % penicillin/streptomycin at 5 % CO_2_.

#### 2.6.1 Cell proliferation

The cell proliferating viability of the blank MA hydrogel, FGF-20 or MAF was detected on Caco-2 cells by CCK-8 assay. Briefly, Caco-2 cells at a density of 1.0 × 10^4^ cells/well were seeded into 96-well plates (Corning, USA) and cultured overnight. Afterward, Caco-2 cells were treated with the fresh culture medium with MA (D-SIS conc. from 1 μg/mL to 500 μg/mL), FGF-20 (FGF20 conc. from 0.5 ng/mL to 250 ng/mL) or MAF (D-SIS conc. from 1 μg/mL to 500 μg/mL; FGF20 conc. from 0.5 ng/mL to 250 ng/mL). After 24 h of culture, cells were washed thrice with PBS and CCK-8 kits (200 μL) was added and incubated for 2 h at 37 °C. The optical density at 450 nm was detected to calculate the cell viability.

#### 2.6.2 Cells migrating test

Cell migrating activity was evaluated by the scratch test. Briefly, Caco-2 cells at a density of 1.0 × 10 ^6^ cells/well were seeded on a 6-well plate. After 72 h of culture, the cell was scratched by a tip. Afterward, cells were treated with the blank MA hydrogel (D-SIS conc. of 200 μg/mL), FGF-20 (100 ng/mL) or MAF (D-SIS conc. of 200 μg/mL; FGF-20 conc. of 100 ng/mL). After 48 h of treatment, the scratch was imaged under an inverted microscope, and the migration rate was calculated.

#### 2.6.3 Immunofluorescent staining of ZO-1, occludin and Claudin-5 of Caco-2 cells

Caco-2 cells at a density of 1 × 10 ^6^ cells per well were seeded in a 12-well plate and cultured for 12 h. Subsequently, cells were treated by H_2_O_2_ (5 mM) for 12 h, followed by addition of MA hydrogel (D-SIS conc. of 200 μg/mL), MAF (D-SIS conc. of 200 μg/mL; FGF-20 conc. of 100 ng/mL) or FGF-20 (100 ng/mL). After 48 h of culture, cells were fixed with 4 % formaldehyde, permeabilized with 0.2 % Triton X-100, and blocked with 3 % bovine serum albumin. Subsequently, cells were stained by primary antibodies and secondary antibodies. Primary antibodies included rabbit polyclonal ZO-1 (AF 5145, 1:1000), rabbit polyclonal Occludin-1 (27260-1-AP, 1:500), or rabbit polyclonal Claudin-5 (AF 5261, 1:500). Goat anti-rabbit IgG Alexa Fluor 488-conjugated secondary antibody was used. Cells was imaged on microscope (TI-S, Nikon) and quantitatively analyzed by Image J software.

### 2.7 *In vivo a*nimal experiments

Adult male SD rats (180–220 g) were purchased from the Laboratory Animals Center of Wenzhou Medical University. All animals were housed and bred in a specific pathogen-free facility with a 12 h light/dark cycle. All rats were acclimated for at least one week before the experiments. All animal experiments were approved by the Institutional Animal Care and Use Committee of Wenzhou Medical University (xmsq 2024-0281).

#### 2.7.1 Constructing TNBS-induced colitis rat model

Colitis rats was induced by rectally perfusing 2,4,6-trinitro-benzenesulfonic acid (TNBS) solution previously described in previous study. Briefly, TNBS in 50 % ethanol (500 μL, 50 mg/kg body weight) was rectally perfused to colon at 6 cm away from the anus of rat. During the whole experiment, the body weight of animals was daily monitored, the disease symptoms (diarrhea, stool and rectal bleeding) was real-time record and disease activity index (DAI) was scored by the method in previous study. When bodies weight loss was reaching to 10 % and DAI scores exceeded 3, the colitis rats’ model was considered successful.

#### 2.7.2 The therapeutic regime

TNBS-induced colitis rats were randomly divided into five groups (5–8 animals per group): (1) TNBS group without therapy (TNBS); (2) FGF-20 solution (5 μg/kg body weight), (3) MAF hydrogel (equivalent FGF-20 at 5 μg/kg body weight); (4) the blank MA hydrogel. Healthy rats (Normal group) were rectally administered with PBS (pH 7.4) as a control. Treatment began on the 4th day after TNBS damage, total six treatments were conducted and these treatments were performed every alternate day. All test samples were rectally administrated and the volume of each test sample every treatment was fixed to 0.5 mL. Drinking water and food were fed during the entire treatment period. At the endpoint, all rats were sacrificed and colons was collected for further histological analysis.

#### 2.7.3 H&E, AB-PAS staining and transmission electron microscopy

Colon tissue was successively fixed, embedded, sliced and dewaxed. The colonic section was stained with hematoxylin and eosin (H&E) or Alcian Blue-Periodic acid Schiff (AB-PAS) and imaged by bright-field microscopy. Besides, the ultra-structure of colon was also observed by transmission electron microscope (TEM). Colon tissues were successively fixed by glutaraldehyde and osmium tetroxide, followed by blocking with uranyl acetate. After dehydration in series of acetone, tissues were embedded in Araldite and coronally sliced. Semi-thin section and toluidine blue staining were performed. Finally, ultrathin sections were observed by TEM apparatus (JEM 1400).

#### 2.7.4 ELISA assays of IL-6, IL-1β, TNF-α

Fresh colon was cut into fine bulk, homogenized in PBS buffer at 4 °C, and centrifuged to collect the supernatants. The levels of IL-6, IL-1β, or TNF-αIL-10 were measured by ELISA kits.

#### 2.7.5 Immunofluorescent staining

The deparaffinized sections were subjected to epitope retrieval and blocked by BSA, followed by incubation with primary antibodies overnight at 4 °C. The sections were then washed with PBS, incubated with secondary antibodies at room temperature, and stained by DAPI. The primary antibodies included rabbit polyclonal ZO-1 (AF5145, 1:400, Affinity®), rabbit polyclonal Occludin-1 (DF7504, 1:200, Affinity®), rabbit polyclonal Claudin-5 (AF5216, 1:300, Affinity®), MUC2 antibody (DF8390, 1:200, Affinity®), CK-18 antibody (10830-1-AP, 1:200, Proteintech®), LGR5 antibody (DF2816, 1:300, Affinity®), or SOX-9 antibody (AF6330, 1:200, Affinity®). The secondary antibodies included donkey anti-rabbit IgG Alexa Fluor 488 (ab150073, 1:1000, Abcam®) or goat anti-mouse IgG TRITC (ab6786, 1:1000, Abcam®). The stained sections were subsequently observed by confocal laser scanning microscopy (Nikon, Ti-E&A1plus). To analyze the proportion of positively stained cells, at least 4 representative areas were measured by ImageJ analysis software.

### 2.8 Statistical analysis

All data are expressed as mean ± standard deviation. Quantitative analysis of the fluorescence intensity was performed using Image-Pro Plus 6.0. Statistical comparisons were performed by analysis of variance (ANOVA) and *t*-test. Statistical analyses were performed using GraphPad Prism 8.0 software.

## 3 Results and discussion

### 3.1 Development and characterization of MAF hydrogel

**Fig. 2:**
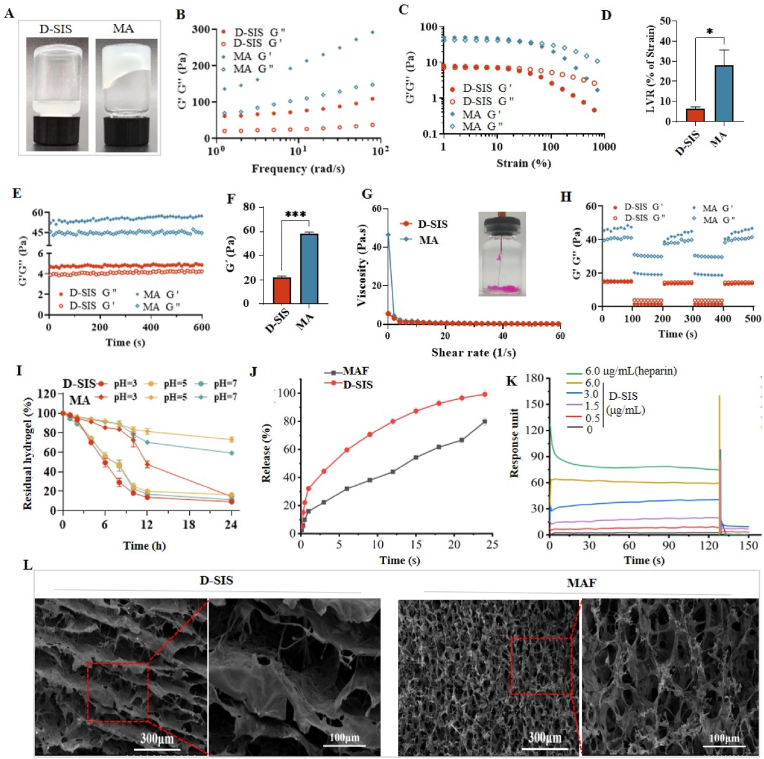
Characteristics of MAF hydrogel: (A) appearance of MA hydrogel; (B) oscillation-frequency sweeps and (C) oscillation-strains sweeps of MA hydrogel or D-SIS liquid; (D) the linear viscoelastic region (LVR) of MA hydrogel or D-SIS liquid; (E) oscillation-time sweeps of MA hydrogel or D-SIS liquid; (F) the quantitative analysis of storage moduli (G′) based on oscillation-time sweeps; (G) the viscosity of MA hydrogel against shear rates (insect: MA hydrogel through 27 G-sized needle); (H) thixotropy of MA hydrogels at low strain (0.1 %) or high strain (100 %); (I) erosion-resistant property of MA hydrogel in excess of artificial colon fluids at 37 °C; (J) *in vitro* release profile of FGF-20 from MAF hydrogel in artificial colon fluids (pH 7); (K) SPR results of D-SIS binding with FGF-20; (L) SEM images of MAF hydrogel or D-SIS.

Porcine small intestinal submucosa (SIS) is a translucent submucosa derived from jejunum. In this study, decellularized SIS (D-SIS) has been prepared and characterized in our previous study [31]. It was demonstrated that the residual DNA content of D-SIS was 10.9 ng/mg dry ECM weight, which was below the commonly accepted threshold (50 ng/mg dry ECM weight). Moreover, the major components of ECM such as Collagen-I, Collagen-III, elastin, fibronectin, laminin and glycosaminoglycans were well retained in D-SIS [31]. Due to its good biocompatibility, biodegradability, and capacity to repairing autologous tissue growth, D-SIS hydrogel has shown great potential in gastrointestinal repair, particularly for ulcerative colitis and colonic anastomotic leakage treatment [34]. However, D-SIS hydrogel usually presented challenges in shape control and relatively weaker mechanical properties, which hindered its potential for clinical translation. Truly, in our study, after D-SIS was digested by pepsin, it formed the viscous and flowable liquid instead of hydrogel. Hyaluronic acid (HA), a naturally occurring glycosaminoglycan (GAG), has been incorporated into ECM hydrogels to improve their mechanical strength while maintaining biological function. In our study, we also observed that HA could significantly enhance the mechanistic strength of D-SIS hydrogel. When the digested D-SIS solution was supplemented with HA, the semi-solid MA hydrogel was formed (Fig. 2A). The rheologic properties of MA hydrogel was further measured. Oscillation-frequency sweeps showed that MA hydrogel exhibited the storage modulus (G′) exceeding loss modulus (G″) while the viscous liquid presented G' < G'' (Fig. 2B). This result further confirmed the hydrogel formation of MA. Moreover, Oscillation-strain sweeps revealed the obvious increase in stiffness of MA hydrogel in comparison with that of D-SIS (Fig. 2C/D). Besides, the mechanic strength of MA hydrogel (G′) was reaching to 50 ± 5 Pa (Fig. 2E/F), which was comparable to that of human gut mucus [35]. The reason why HA enhance the mechanistic strength of D-SIS hydrogel might be attributed to the two aspects. On the one hand, high-molecular-weight HA increases hydrogel viscosity by forming entangled networks with D-SIS fibrillar proteins (*e.g.,* collagen, elastin) [36]. On the other hand, the polyanionic nature of HA strengthens the hydrogel by forming ionic bridges with D-SIS proteins (*e.g.*, collagen, growth factors), increasing the mechanical modulus of hydrogel [37].

Injectability refers to the ability of a hydrogel to be extruded through a needle or catheter, which depends on its rheological properties (*e.g.,* viscosity, shear-thinning behavior). The viscosity of MA hydrogel against shearing rate was further measured to evaluate its shear-thinning behavior. As shown in Fig. 2G, the viscosity of MA hydrogel at shear rate of 0.1 s^−1^ was 45 Pa s, which obviously decreased as shear rates increased. This indicated MA hydrogel had good shear-thinning behavior. Moreover, MA hydrogel was easily injected through a 27 G-sized needle. The rapid recovery of storage/loss moduli was also observed for MA hydrogel at alternative high/low strains (Fig. 2H). These results suggested that MA hydrogel exhibited the good injectability and thixotropy, which might be beneficial for rectal administration. Moreover, the loading of FGF-20 did not affect these characteristics of MA hydrogel owing to its very low drug loading amount in MAF hydrogel.

*In vitro* erosion of MA hydrogel was investigated in artificial colon fluid. MA hydrogel was slowly eroded in pH 5–7 artificial colon fluid within 24 h (Fig. 2I). More than 60 % of MA hydrogel was still residual, whereas the viscous D-SIS liquid was completely dissolved in artificial colon fluids within 12 h. We further detect the release profile of FGF-20 from MAF hydrogel in pH 7 artificial colon fluid and results were shown in Fig. 2J. As expected, FGF-20 was slowly released from MAF hydrogel within 24 h. By contrast, most of FGF-20 (>80 %) was released from the viscous D-SIS liquid within 12 h. The release-sustained behavior might be due to the combination of the erosion-resistant characteristic and the affinitive interaction of FGF-20 with D-SIS. This hypothesis was confirmed by the concentration-dependent response of SPR (Fig. 2K). The response signal of FGF-20 to D-SIS was near to that in heparin at the same concentration, indicating the strong interaction between FGF-20 and D-SIS. FGF-20 is a key paracrine growth factor in various tissues, which is mainly stored as inactivated form in extracellular matrix [38]. FGF-20 contains a heparin-binding domain, allowing high-affinity interactions with D-SIS-associated heparan sulfate [39]. Besides, the nanoporous structure of D-SIS (fibrillar collagen, elastin) might physically trap FGF-20 via hydrophobic interactions, which was similarly observed in previous study [40]. Alternatively, the microscopic morphology of MA hydrogel was imaged by scanning electronic microscopy and results were shown in Fig. 2L. The thin fibrous morphology was observed for SEM image of D-SIS while MA hydrogel presented the highly porous network of thin, intertwined fibers. The physical entanglement between D-SIS and HA might be implicated in MA hydrogel, which rendered it with the erosion-resistant property. Collectively, an injectable MAF hydrogel was prepared by supplementing D-SIS with HA, which might be suitable for the rectal delivery of FGF-20.

### 3.2 MAF hydrogel repaired the injured Caco-2 cells

**Fig. 3:**
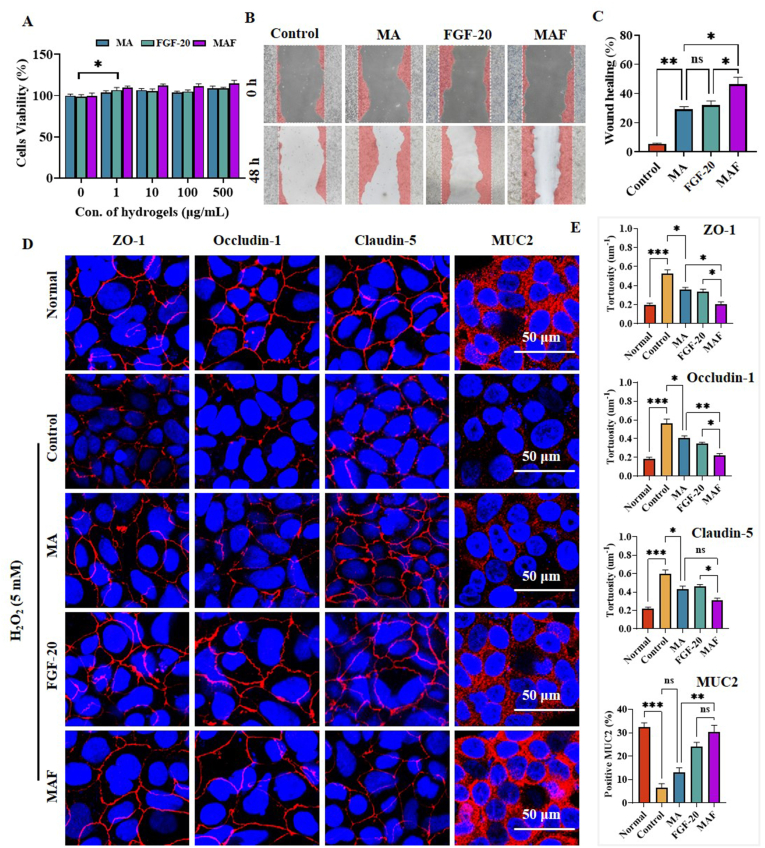
The repairing effect of MAF hydrogel on the injured Caco-2 cells: (A) cell viability of Caco-2 cells after 24 h of treatment; (B) the wound healing of Caco-2 cells after 48 h of treatment; (C) the quantitative analysis of wound healing based on the scratch test; (D) immunofluorescent (IF) staining of tight junctions proteins (ZO-1, Claudin-5, and Occludin-1) and mucus protein (MUC2) of Caco-2 cells; (E) quantitative analysis of tortuosity based on IF staining of tight junctions and mucus protein (∗∗∗p < 0.001, ∗∗p < 0.01, ∗p < 0.05, n = 3).

The biocompatibility of the blank MA hydrogel was firstly investigated on Caco-2 cells and results were shown in Fig. 3A. No toxicity of MA hydrogel against Caco-2 cells was observed even its concentration was as high as 500 μg/mL. Moreover, the cell proliferation was slightly enhanced by MA hydrogel at low concentration of 1 μg/mL. Subsequently, the bioactivity of FGF-20 in MAF hydrogel was detected by cell proliferation. Interestingly, the cell proliferating activity of FGF-20 in MAF hydrogel was well retained, which was significantly higher than that of free FGF-20 at same concentration. The similar results were observed by cell scratch test (Fig. 3B). After 48 h of treatment, about 50 % of wound was healed by MAF hydrogel while free FGF-20 healed ca. 30 % wound (Fig. 3C). These suggested that the bioactivity of FGF-20 was well protected by MA hydrogel. The similar phenomenon that FGF-2 was stabilized by ECM-based scaffolds was reported in previous study [41]. ZO-1 (Zonula Occludens-1), Claudin-5, and Occludin-1 are key components of tight junctions, and their morphology and organization are critical for barrier integrity. In this study, we first treat Caco-2 monolayers with H_2_O_2_ (5 mM) to damage the epithelial barrier. As shown in Fig. 3D, the expression of these proteins was substantially down-regulated and their morphology was obviously deformed after injury in comparison with normal Caco-2 cells. The morphology and tortuosity of these proteins can provide valuable insights into barrier function and the effects of experimental treatments [42]. After treatment with MA, FGF-20 or MAF hydrogel, these proteins expression was restored and their distorted morphology was largely normalized. Especially, MAF treatment resulted in the comparable tortuosity with that of normal cells (Fig. 3E). The intestinal mucus barrier is critical for protecting epithelial surfaces from pathogens, toxins, and mechanical stress. Caco-2 cells, a well-established model of intestinal epithelium, expressed mucus-relevant protein (MUC2), which was greatly damaged in presence of H_2_O_2_ (Fig. 3D). FGF-20 played essential roles in mucus secretion and epithelial repair, making it relevant for intestine regeneration. Following FGF-20 or MAF treatment, MUC2 expression was greatly restored. Moreover, the most restoration of MUC2 was observed in MAF group, which was near to the comparable level in normal group (Fig. 3E). These results demonstrated that MAF could effectively repair the integrity of the epithelial barrier.

### 3.3 MAF hydrogel enema ameliorated colitis in rats

**Fig. 4:**
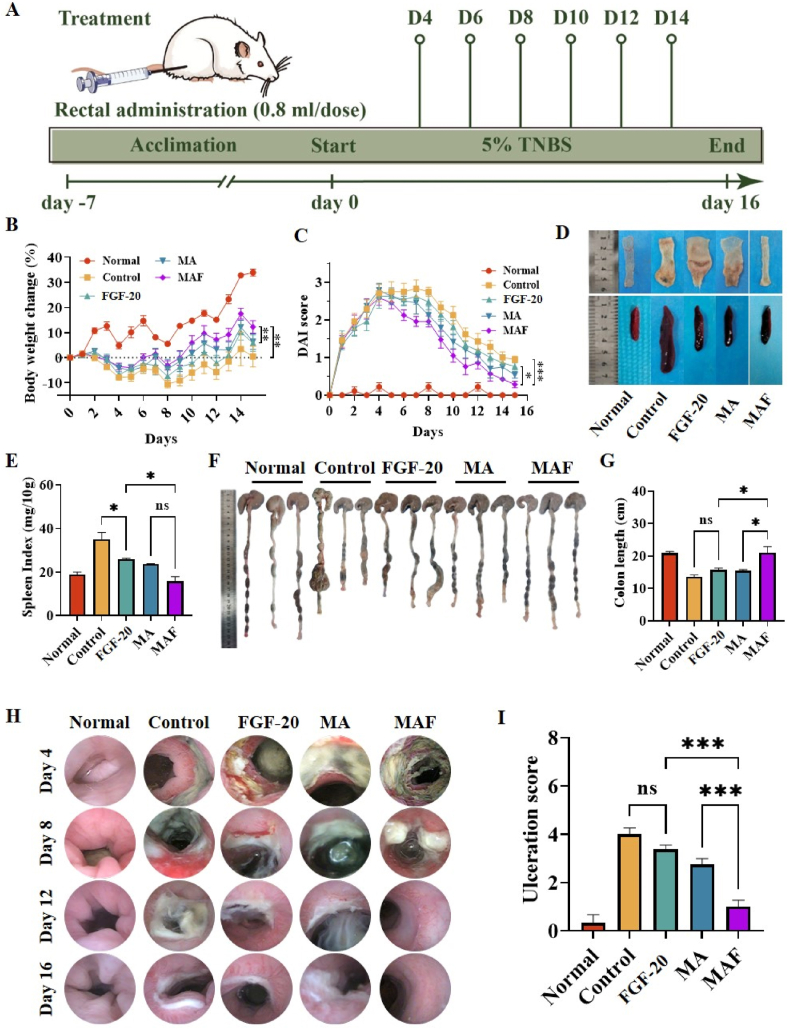
MAF hydrogel enema ameliorated colitis in rats: (A) establishment of colitis model in rats and the experimental regime; (B) body weight loss and (C) disease activity index (DAI) of colitis rats during the experimental period; (D) the spleen index of colitis rats; (E) the representative images of colon and (F) the colon length; (G) the representative colonoscopy images of rats and (H) ulceration scores based on colonoscopy images (∗∗∗p < 0.001, ∗∗p < 0.01, ∗p < 0.05).

A rat model of ulcerative colitis (UC) was established by administering TNBS via enema according to the experimental regime in Fig. 4A. Rats in the TNBS group exhibited significant weight loss and elevated DAI scores (Fig. 4B/C), confirming the successful induction of colitis. Colitis rats were treated rectally with MAF, MA, or FGF-20 once every three days and total six treatments was performed (Fig. 4A). Treatment with MAF, MA, or FGF slowed the weight loss trend and reduced DAI scores, indicating therapeutic efficacy. Among the treatments, MAF hydrogel demonstrated the most significant improvement (Fig. 4B/C). At the endpoint of the study, rats were euthanized, and their colons and spleens were collected for further analysis. Treatment with MAF, MA, or FGF-20 reduced spleen index, with MAF showing the most pronounced effect (Fig. 4D/E). Decreasing spleen index in MAF group reflected the pronounced amelioration of the inflammatory response associated with ulcerative colitis. Colitis rats in the TNBS group exhibited significant colon shortening and swelling. FGF-20 or MA treatment partially improved these parameters, while MAF restored colon length and morphology to near-normal levels (Fig. 4F/G). Besides, small animal endoscopy revealed that the severe mucosal erosion, bleeding, and fibrosis of colitis rats was largely reversed by MAF treatment (Fig. 4H). By contrast, FGF-20 or MA treatment resulted in the partial improvement of these pathological markers. Moreover, the significant fibrosis in these two groups were still visible. Besides, ulcer scores based on vascular morphology, ulcer size, and rectal stenosis, were further assessed, which were highest in the TNBS group. Treatment with FGF-20 or MA resulted in slight reductions in ulcer scores, while MAF treatment led to a significant reduction, demonstrating superior therapeutic efficacy (Fig. 4H/I). FGF-20 has been demonstrated to be effective to combat the intestinal inflammation and reduce the severity and extent of mucosal damage in DSS-colitis model [43]. The limited therapeutic effect of FGF-20 solution enema on colitis mice might be due to two reasons. First, the dose of FGF-20 (5 μg/kg body weight) was slowed approximately three orders of magnitude in comparison with that in previous study (5 mg/kg body weight) [43]. Second, FGF-20 was administrated via solution enema to colitis colon, rendering it difficulty to adhere on the wriggling colon. By contrast, MAF in this study emerged as a highly effective therapeutic agent, outperforming both MA and FGF-20 alone. The superior efficacy of MAF can be attributed to its dual functionality. On the one hand, MAF likely formed a barrier over the damaged mucosa, preventing further injury and promoting healing. On the other hand, MAF not only enhanced FGF-20 stability but also facilitated its controlled release, which was very helpful for gut epithelial repair and regeneration.

### 3.4 Repairing tissue morphology and alleviating the mucosal inflammation

**Fig. 5:**
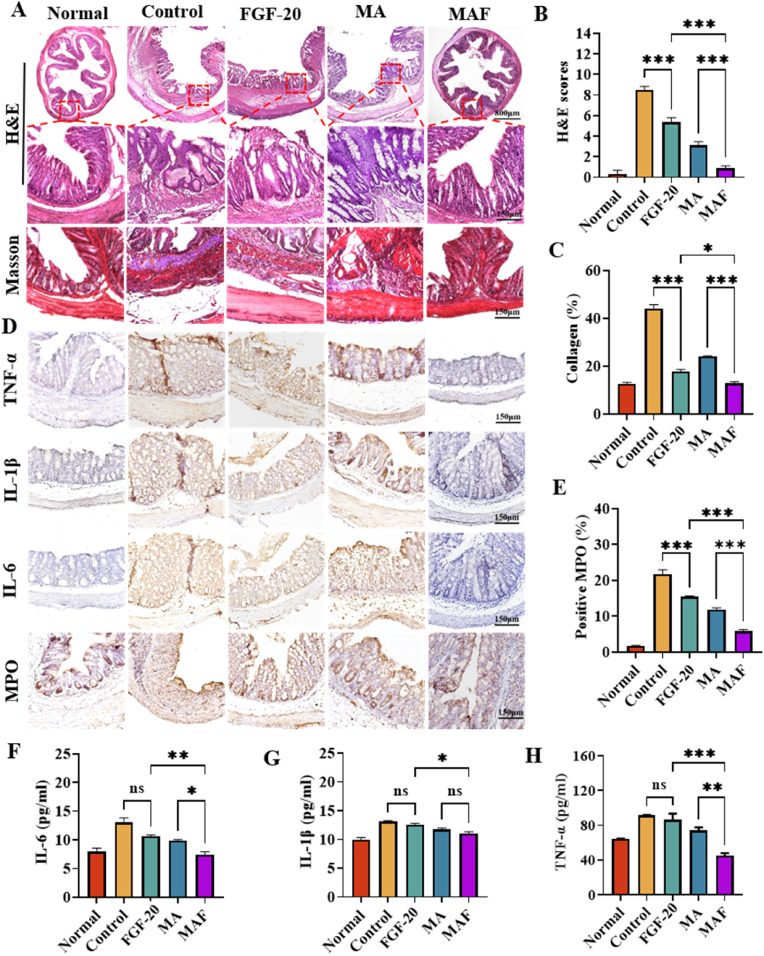
Alleviating the gut mucosal inflammation: (A) H&E and Masson staining; (B) Histological scores based on H&E staining and (C) quantitative collagen analysis based on Masson staining; (D) ICH staining of IL-6, TNF-α, IL-1β or MPO; (E) quantitative statistics of MPO based on ICH staining; (F–H) ELISA assay of TNF-α, IL-1β and IL-6 (∗∗∗p < 0.001, ∗∗p < 0.01, ∗p < 0.05).

The tissue morphology of the colon was firstly evaluated by hematoxylin and eosin (H&E) staining. As shown in Fig. 5A/B, the TNBS group exhibited severe pathological changes, including crypt destruction, goblet cell atrophy, and extensive inflammatory cell infiltration, which are consistent with the typical features of ulcerative colitis [[44], [45], [46]]. Treatment with FGF-20 or MA alone partially alleviated these pathological alterations. In contrast, MAF treatment significantly reversed these pathological symptoms, restoring the colon morphology to a level comparable to that of healthy rats. Alternatively, ulcerative colitis is often associated with intestinal fibrosis due to dysregulated extracellular matrix remodeling [47]. Masson's trichrome staining revealed extensive collagen deposition in the colon tissues of the TNBS group (Fig. 5A/C). Treatment with MAF, MA, or FGF-20 attenuated intestinal fibrosis to varying degrees, with MAF showing the most pronounced inhibitory effect. Furthermore, the development and progression of colitis involved a series of immune-inflammatory activation. The expression of pro-inflammatory cytokines, including TNF-α, IL-1β, and IL-6, was significantly elevated in the colonic mucosa of the TNBS group (Control) (Fig. 5D). Besides, myeloperoxidase (MPO), a marker of neutrophil infiltration, was also significantly increased in this group (Fig. 5E). FGF-20 or MA treatments did not significantly reduce the level of TNF-α, IL-1β, IL-6 and MPO. But MAF treatment markedly suppressed the levels of these inflammatory cytokines (Fig. 5 D). The similar results were also observed by ELISA assay of TNF-α, IL-1β, IL-6 (Fig. 5F–H), demonstrating MAF had the potent anti-inflammatory effects.

### 3.5 Restoring tight junction proteins in colitis

**Fig. 6:**
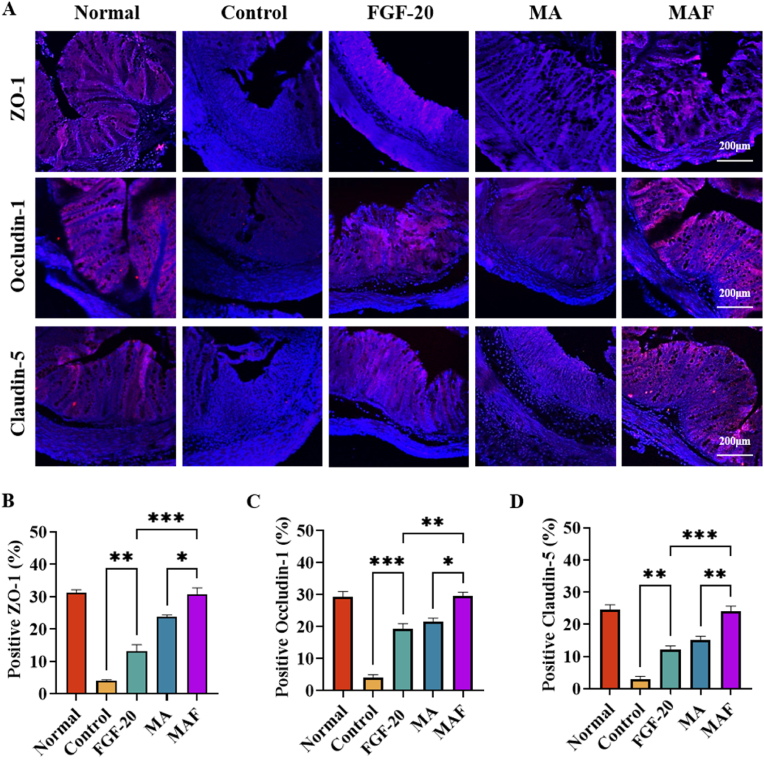
Restoring tight junction proteins in colitis: (A) the immunofluorescence staining of Tight junction (TJ) proteins (ZO-1, occludin-1, and claudin-5) of colon tissues; (B–D) the quantitative analysis of ZO-1, Occludin-1 and Claudin-5 based on the immunofluorescence staining.

The gut mucosal barrier, comprising the mucus layer and tight junction proteins, plays a pivotal role in maintaining intestinal homeostasis and preventing inflammation [48]. Tight junction (TJ) proteins, including ZO-1, Occludin-1, and Claudin-5, are critical for maintaining the structural and functional integrity of the gut epithelial barrier [49]. In ulcerative colitis, the expression and localization of TJ proteins are often disrupted, leading to "leaky gut" syndrome, characterized by increased intestinal permeability and heightened immune activation. Truly, in this study, results of immunofluorescence staining showed that the expression of TJ proteins was greatly down-regulated in a TNBS-induced ulcerative colitis rat model (Fig. 6A). The results revealed the severe impairment of the gut barrier. Treatment with FGF-20 or MA alone partially restored the expression of TJ proteins, suggesting a moderate protective effect on the gut epithelial barrier. However, MAF treatment significantly reversed the downregulation of TJ proteins (Fig. 6B–D), showing the best effect of restoring their expressions. This robust restoration of TJ proteins for MAF treatment highlights its potential to repair the gut epithelial barrier and mitigate the pathological features of colitis. The restoration of TJ proteins by MAF is likely mediated through multiple mechanisms, including the suppression of pro-inflammatory cytokines, reduction of oxidative stress, and modulation of signaling pathways involved in TJ assembly and maintenance.

### 3.6 Restoring the mucus secretion of goblets cells

**Fig. 7:**
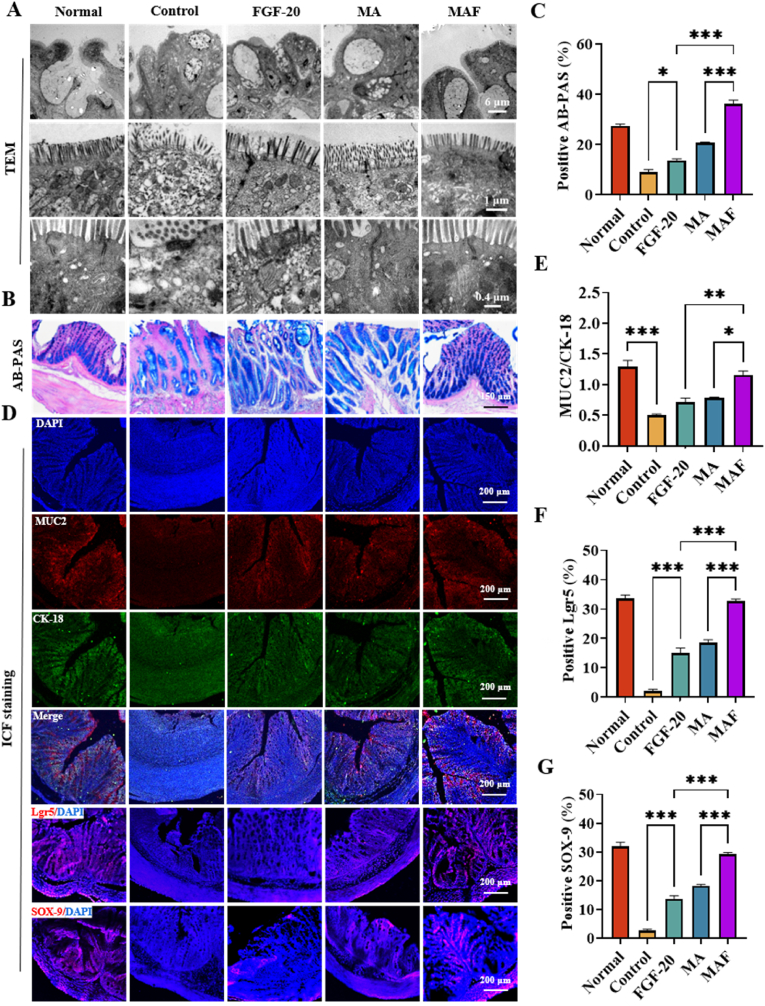
Restoring the mucus secretion of goblets cells: (A) TEM ultrastructure of colonic tissues; (B) AB-PAS staining of the glycosylated mucus and (C) its quantitative analysis; (D) immunofluorescence staining (IF) of MUC 2, CK 18, Lgr 5 and SOX-9; (E) quantitative analysis of MUC2^+^/CK18^+^ (functional globets) staining; the quantitative analysis of Lgr 5 (F) and SOX-9 (G) (∗∗∗P < 0.001, ∗∗P < 0.01, ∗P < 0.05).

The mucus layer, primarily composed of mucins secreted by goblet cells, forms a physical and biochemical barrier that protects the gut epithelium from direct contact with luminal contents. In ulcerative colitis, the mucus layer is often disrupted, leading to reduced thickness and altered composition. This impairment compromises the barrier's ability to trap and exclude pathogens, contributing to increased epithelial exposure to microbial antigens and subsequent inflammation. Furthermore, goblet cell depletion, a hallmark of colitis, exacerbates mucus layer dysfunction, further weakening the mucosal defense system. In this study, the ultrastructure of colonic tissues was further examined using transmission electron microscopy (TEM). Goblet cells were severely collapsed, microvilli were loosely detached and tight junctions was greatly impaired in TNBS-induced rats (Control) (Fig. 7A). These ultrastructural abnormalities are consistent with the disruption of the intestinal epithelial barrier, a hallmark of ulcerative colitis (UC). Treatment with FGF-20 or MA alone partially improved these pathological changes. By contrast, MAF treatment resulted in the most significant restoration, *e.g.,* goblet cells were normalized, microvilli were tightly aligned and tight junctions was greatly restored.

Glycosylated mucins were secreted by goblet cells and form a protective mucus layer over the gut epithelium. Alcian Blue-Periodic Acid-Schiff (AB-PAS) staining revealed that mucus secretion was significantly reduced in the TNBS group (Fig. 7/C), indicating impaired mucus barrier function. This defect was reversed after treatment with MAF, MA, or FGF-20. Among these treatments, MAF showed the most pronounced effect. Furthermore, immunofluorescence staining for MUC-2 (a major secretory mucin) and CK-18 (a marker of epithelial cells) confirmed the restoration of mucin distribution in the colon, supporting the AB-PAS findings (Fig. 7D/E). These results suggest that MAF effectively promotes mucus barrier repair, which is essential for protecting the intestinal epithelium from luminal pathogens and toxins.

The functional maintenance of the intestinal epithelial barrier relies on the self-renewal capacity of intestinal stem cells (ISCs), which reside in the crypts and give rise to newborn epithelial cells [50]. To investigate the role of ISCs in epithelial repair, the expression of ISC markers, Lgr5 and SOX-9, was analyzed. In TNBS-induced rats, the expression of both markers was significantly reduced, indicating the impairment of ISC activity. Treatment with FGF-20 or MA partially reversed this defect, but MAF treatment led to a significant upregulation of Lgr5 and SOX-9 expression (Fig. 7D/E-F). These findings suggest that MAF promotes intestinal epithelial repair and mucus barrier restoration by enhancing the differentiation and proliferation of ISCs.

## 4 Conclusions

In summary, an injectable MAF hydrogel was successfully developed by supplementing D-SIS with HA, addressing the limitations of D-SIS in shape control and mechanical strength. MAF hydrogel exhibited excellent rheological properties, erosion resistance, and sustained drug release, making it a promising candidate for the rectal delivery of FGF-20 in the treatment of ulcerative colitis. Besides, MAF hydrogel demonstrated excellent biocompatibility and effectively preserved the bioactivity of FGF-20, enhancing its ability to promote epithelial cell proliferation and wound healing. Furthermore, MAF hydrogel significantly restored the expression and morphology of tight junction proteins, repairing the integrity of the epithelial barrier in an H_2_O_2_-induced Caco-2 monolayers. Animal experiments demonstrated that MAF hydrogel had superior therapeutic efficacy in a TNBS-induced UC model, outperforming both MA and FGF-20 alone. Moreover, MAF treatment led to the restoration of colonic morphology, reduction of intestinal fibrosis, and suppression of pro-inflammatory cytokines and neutrophil infiltration. More important, both mucus and the epithelial barriers in colitis rats were well restored by MAF treatment. These effects are likely mediated through multiple mechanisms, including the promotion of ISC differentiation, suppression of inflammatory signaling pathways, and stimulation of mucus production. In TNBS-induced colitis rats, we did not observe any adenomas or abnormal plaque in whole colon tissues after four times treatment with MAF at FGF-20 dose of 5 μg/kg body weight. Besides, there existed no signs of toxicity in major organs (heart, liver, lung and kidney) after MAF treatments (Fig. S1). These suggested MAF might be well tolerated in colitis animals. In future, we will further confirm its safety in AOM/DSS-induced colitis-relevant tumor mice. Moreover, the molecular mechanisms underlying MAF's effects would be also further elucidated.

## CRediT authorship contribution statement

**Minmin Wang:** Methodology, Investigation, Conceptualization. **Dingwei Li:** Methodology, Investigation. **Shenyuan Ouyang:** Investigation, Funding acquisition. **Bingjie Tong:** Methodology, Formal analysis. **Yumo Chen:** Methodology, Investigation. **Bingyu Ding:** Methodology, Investigation. **Jie Wang:** Methodology, Investigation. **Zhijiang Jiang:** Methodology. **Helin Xu:** Supervision, Project administration, Funding acquisition, Conceptualization. **Sunkuan Hu:** Supervision, Funding acquisition, Conceptualization.

## Declaration of competing interest

We would like to submit the enclosed manuscript entitled “Hydrogel derived from decellularized pig small intestine submucosa boosted the therapeutic effect of FGF-20 on TNBS-induced colitis in rats via restoring gut mucosal integrity” by Minmin Wang, Dingwei Li, Shenyuan Ouyang, Bingjie Tong, Yumo Chen, Bingyu Ding, Jie Wang, Zhijiang Jiang, Helin Xu, Sunkuan Hu, which we wish to be considered for publication in “Materials Today Bio”. No conflict of interest exits in the submission of this manuscript, and manuscript is approved by all authors for publication. I would like to declare on behalf of my co-authors that the work described was original research that has not been published previously, and not under consideration for publication elsewhere, in whole or in part.

## Data Availability

Data will be made available on request.
