# Supplementary material for: Hydrogel derived from decellularized pig small intestine submucosa boosted the therapeutic effect of FGF-20 on TNBS-induced colitis in rats via restoring gut mucosal integrity
Source: Mater Today Bio. 2025 Apr 20;32:101783. doi: 10.1016/j.mtbio.2025.101783 (PMC12049826; doi:10.1016/j.mtbio.2025.101783)
Supplement: Multimedia component 1 [file mmc1.docx]

Supporting information


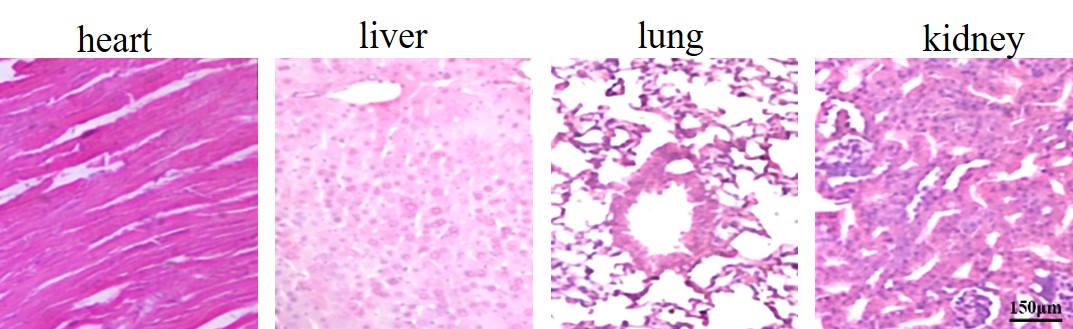


Fig S1 H&E staining of heart, liver, lung and kidney from MAF-treated colitis rats
